# The Role of Endoscopic Ultrasound in Cardiology: Clinical Applications and Future Perspectives, a New Era of Minimally Invasive Cardiovascular Diagnosis and Intervention

**DOI:** 10.3390/jcm15135006

**Published:** 2026-06-26

**Authors:** Abdelrahman Elhakim, Mohammad El Garhy, Philip Sauter, Ahmed Abdelsalam, Mohamed Elhakim, Osama Bisht, Mohammed Saad

**Affiliations:** 1 Internal Medicine Department, Schoen Hospital Neustadt in Holstein, Am Kiebitzberg 10, 23730 Neustadt in Holstein, Germany; psauter@schoen-klinik.de; 2 Internal Medicine Department 1, Am Klinikum 1, 07747 Jena, Germany; mohammad.elgarhy@med.uni-jena.de; 3 Cardiology Department, Sana Hospital Hof, Eppenreuther Street 9, 95032 Hof, Germany; abdelsalamahmed25@gmail.com; 4 Intensive Care Medicine Department, The Royal Prince Alfred Hospital, 50 Missenden Road, Camperdown, Sydney NSW 2050, Australia; myelhakim@hotmail.com; 5 Cardiology Department, Coswig Heart Center, Lerchenfeld 1, 06869 Coswig (Anhalt), Germany; obisht@gmail.com; 6 Cardiology Department, Sana Hospital Coburg, Ketschendorfer Street 33, 96450 Coburg, Germany; mohsaazak@hotmail.com

**Keywords:** endosonographic ultrasound, transesophageal echocardiography, angiosarcoma, left atrial appendage thrombus, mitral valve, tumor, space-occupying lesion

## Abstract

**Background**: Ultrasonography during medical procedures allows for real-time assessment of the target structure. This design feature could be more advantageous than radiological “snapshot” imaging. Technically, the image resolution of endoscopic ultrasound (EUS) is noticeably higher than that of transesophageal echocardiography (TOE). The benefits of investigating cardiovascular structures using this mode of high-resolution EUS are unknown. **Materials and Methods**: We present clinical applications in the diagnosis of cardiovascular structures, demonstrated during routine gastrointestinal endosonographic procedures. In some cases, these diagnoses led to changes in management strategies. **Results and Discussion**: The introduction of high-resolution EUS into cardiology allows for the accurate definition of variable cardiovascular anatomy and early detection of asymptomatic cardiac pathologies. It also prevents double investigations for patients and operators, reduces the risk of esophageal trauma, and highlights the benefits of interdisciplinary teamwork. In addition, the high spatial resolution of EUS facilitates detailed tissue characterization for guiding biopsies, thereby extending the applicability of elastography across various echocardiographic domains. Moreover, the precision of using EUS when targeting and inserting a needle into adjacent organs could facilitate the development of EUS indications for cardiac-based interventions. **Conclusions**: The use of EUS in cardiology provides high-resolution real-time assessments of cardiovascular anatomy and may facilitate the development of EUS indications for cardiac-based interventions. However, large cohort studies are required.

## 1. Introduction

Ultrasonography (US) in cardiovascular medicine allows for the real-time assessment of structures during medical procedures. A panoramic view of dynamic behavior over time can be obtained by the digital stitching of multiple ultrasound images into a single broader image.

This design feature could be more advantageous than radiological “snapshot”-view imaging. Its availability, low cost, and lack of ionizing radiation make US the most applicable imaging tool for the assessment of cardiovascular and gastrointestinal structures [1].

However, sonographic devices have limitations related to their use, and they yield very poor image quality when there is bone, fat, or gas between the transducer and the organ of interest. The vast differences in acoustic impedance and depth penetration can restrict the sound beam. Thus, the accuracy of diagnoses may be limited, depending on the frequency of imaging [1].

These limitations can be minimized by using transesophageal echocardiography (TOE), which may provide a better image resolution due to the absence of intervening air or bone.

Endoscopic ultrasound (EUS) is recommended for use in the current guidelines regarding cancer management, as it provides images of the highest possible resolution during the local staging of gastrointestinal tumors [2]. In addition, the high spatial resolution of EUS facilitates detailed tissue characterization and guiding biopsies, showing the broader applicability of elastography across various echocardiographic domains.

EUS’s precision in targeting and inserting needles into adjacent organs, a well-established procedure in daily gastrointestinal interventions, could facilitate the performance of transesophageal extra- and intracardiac interventions under direct EUS guidance. The benefits of using this diagnostic and therapeutic tool in investigating cardiovascular disease (CVD) and related structures are unknown. The introduction of EUS in CVD may help management in high-risk, vulnerable, and non-operable patients by facilitating both the diagnosis of the underlying pathology and minimal therapeutic intervention through fine-needle aspiration biopsy. However, more large studies are still required to assess the safety and efficacy of EUS applications in CVD. In addition, a dedicated overview of cardiovascular applications of EUS is needed, as previous publications have focused on its applications in gastroenterology.

## 2. Materials and Methods

**Figure 1:**
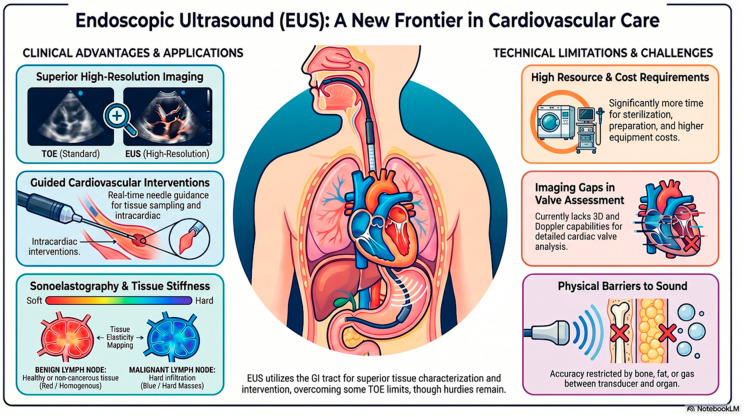
Summary figure of endoscopic ultrasound potential indications, advantages and disadvantages in cardiovascular medicine. Image created using NotebookLM, Google, April 2026.

To outline the difference between echocardiographic and endosonographic ultrasound, the main diagnostic features of both modalities are summarized in Table 1, along with descriptions of how they work (Figure 1).

### 2.1. Endoscopic Ultrasound in Focus

**Figure 2:**
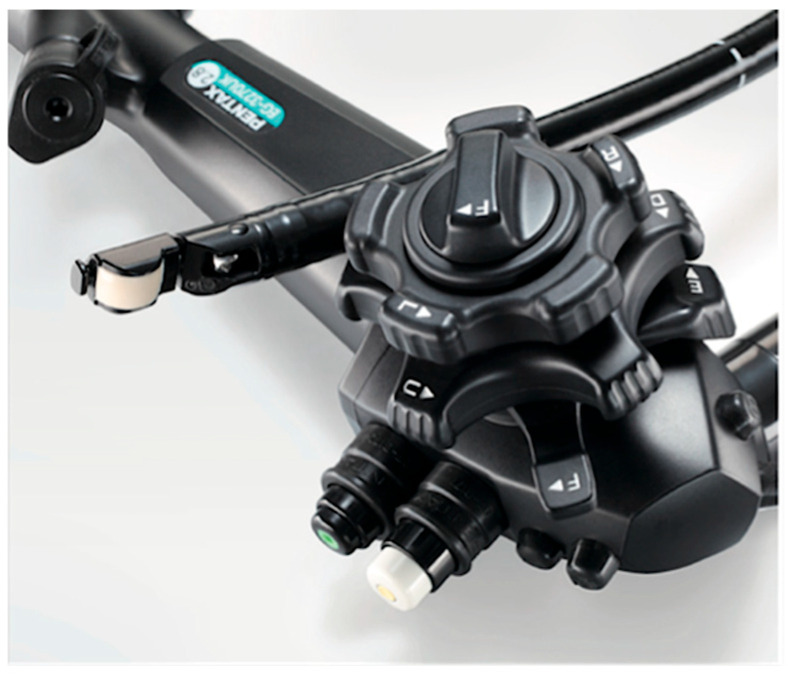
The main features of endoscopic ultrasound EG-3270UK, (Hitachi Medical Systems Europe, Sumpfstraße 13, 6300 Zug, Switzerland, now belonging to Fujifilm), at the proximal and distal ends of the probe [11].

**Figure 3:**
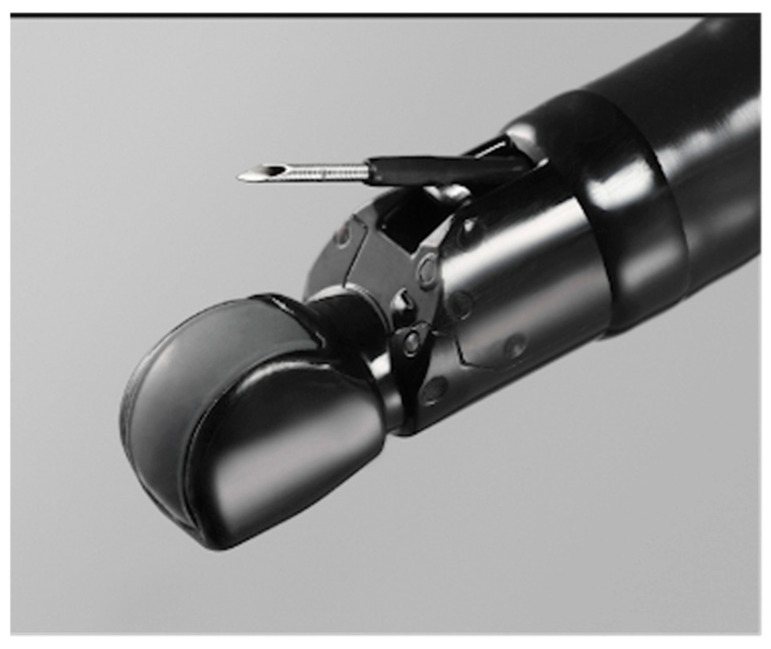
The main features of endoscopic ultrasound Fujifilm EG-740UT curved linear with optimized material elasticity to facilitate advanced force transmission, a short bending radius to facilitate access precision of the targeted area, optimized forward-viewing visualization and Fujifilm G-Lock elevator locking mechanism (19).

**Figure 4:**
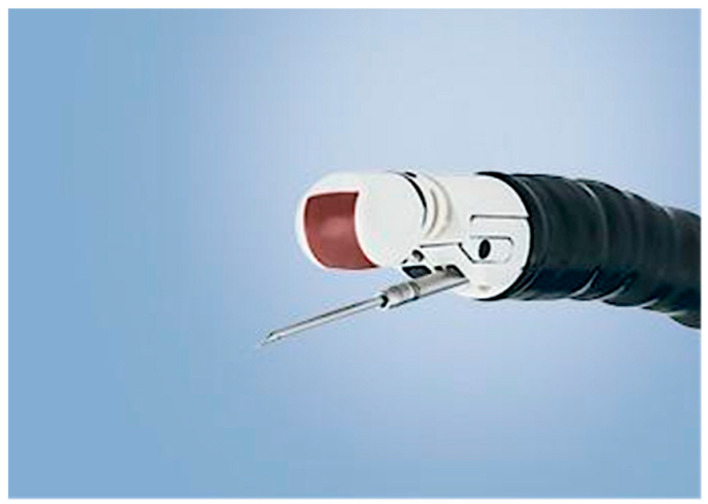
The main features of Olympus TGF-UC180J, Olympus Europa SE and Co., KG, Wendenstraße 20, 20097 Hamburg, Germany, linear ultrasound endoscope for interventional EUS procedures with extremely short distal end, wide angulation, straight channel port and auxiliary water channel. The EZ Shot 3 plus FNB needle offers access to lesions in difficult locations, enhanced echogenicity, and reduced puncture force in 19 G, 22 G, and 25 G configurations to suit preferences in a variety of clinical settings (20).

EUS allows simultaneous endoscopic and ultrasound imaging. It involves an ultrasound processor connected to either a radial or linear transducer attached to the tip of an endoscope. These scopes are then connected to standard video processors [2]. Three major manufacturers, Hitachi (Hitachi Medical Systems Europe, Sumpfstraße 13, 6300 Zug, Switzerland, now belonging to Fujifilm) (Figure 2), Fujifilm Endoscopy (Fujifilm Europe GmbH, Balcke-Dürr-Allee 640882 Ratingen, Germany) (Figure 3), and Olympus (Olympus Europa SE and Co., KG, Wendenstraße 20, 20097 Hamburg, Germany) (Figure 4), produce ultrasound processors, and their products are compatible with different types of EUS [12].

An endoscope comprises (1) an image transmission system that sends images out of the body, (2) a bending mechanism that deflects the endoscope’s tip, (3) channels for the ingress of air to inflate the body cavity and create suction for the removal of fluids, (4) water to wash the objective lens and aid the introduction of biopsy forceps, and (5) a lighting system attached to an external light source [2,12].

Furthermore, the types of EUS differ in terms of US tip design, flexibility, and the system for controlling balloon inflation at the tip. The possibility of balloon inflation before insertion may reduce contact injury and severe discomfort or pharyngeal pain in the patient.

There are two basic echoendoscope designs:

1. Radial EUS is used only for diagnostic purposes and can produce Doppler, color flow, elastosonography and contrast-enhanced endosonographic imagery. It is mainly used for luminal imaging and the evaluation of the layers of the wall of the GI tract, pancreas, and biliary system;
2. Linear array EUS probes (used for both diagnostic and therapeutic purposes) provide several advantages, including improved maneuverability. In addition, the inclusion of bend points at the distal end of the endoscope allows for the precise control of the angle of exit of the EUS needle (or another device) from the working channel, with simultaneous visualization and treatment of the target lesion [2]. The linear array is preferred in most gastrointestinal interventions due to these benefits [18] (Figure 2).

The main diagnostic and therapeutic features of both modalities are summarized in Supplementary Table S1.

### 2.2. Endoscopic Ultrasound: Standard Views and Methods for Looking at Cardiovascular Structures

In general, the ultrasound frequency is adjustable in the range of 5–13 MHz, and the local resolution is excellent to a depth of 100 mm, depending on the tissue impedance and manufacturer [19,20].

After local pharyngeal anesthesia, a smooth advance without great resistance is of the utmost importance to prevent iatrogenic tissue injuries and complications. The EUS probe (viewing angle 120°) advances transesophageally with reference to anatomic landmarks on the sonographic output interpreted by an experienced operator. Notably, all radial EUS scopes have a 360° viewing angle—linear echoendoscopes made by Olympus have a 180° viewing angle, and the new “J” generation of Hitachi (now Fujifilm) scopes have a 150° angle [19,20].

After passing through the oral cavity, the first anatomic landmarks that can be seen are the right carotid artery and the adjacent thyroid lobe (C4 level). After this point, the probe is advanced with the application of moderate pressure (insufflation of carbon dioxide can ease this) until it passes the upper esophageal sphincter (C6 level). Then, it is further advanced along the superior vena cava, ascending aorta, and aortic arch (T4 level) [14]. At the T4–T8 level within the esophagus, the following cardiac structures can be examined.

Around T5/6, the right pulmonary artery and the left atrium can be seen.

If the transducer is turned to the right at the level of the T4/5 aorta, the azygos vein can also be seen and can be traced to its point of continuity with the vena cava. In addition, due to the proximity of the posterior mediastinum to the esophagus and the design of the EUS, these cardiovascular structures can be technically accessed using the EUS. However, the endosonographic probe is not capable of planar rotation, which limits the assessment of different valvular planes.

Supplementary Table S2 summarizes the main features of the Fujifilm EG-740UT, and Supplementary Table S3 summarizes the main features of the TGF-UC180J linear EUS [1].

In this article, we demonstrate an EUS-based cardiovascular anatomy. Symptomatic, silent, and accidental pathologies have been detected and investigated using EUS as a diagnostic tool during routine gastrointestinal investigations. In some cases, the findings induced a change in management strategy [14]. The EUS devices used were Pentax EG-3870 UTK-EUS and EG-3270UK-EUS (Julius-Vosseler-Str. 104, 22527 Hamburg, Germany) [4], while the TOE was performed using a Philips Epic 7 (Veenpluis 4-6, 5684 PC Best, The Netherlands) [7].

### 2.3. Clinical Applications of EUS in Cardiovascular Medicine

The use of EUS in daily clinical practice is exclusively indicated for diagnostic and therapeutic purposes in gastrointestinal disorders, particularly tumor diagnosis and staging. On the other hand, the use of EUS in CVD is limited to clinical experiences and a few case reports.

### 2.4. EUS-Guided Cardiovascular Diagnostics, Clinical Observations, Anecdotal Experience, Technical Feasibility Reports, and Future Perspectives

**Figure 5:**
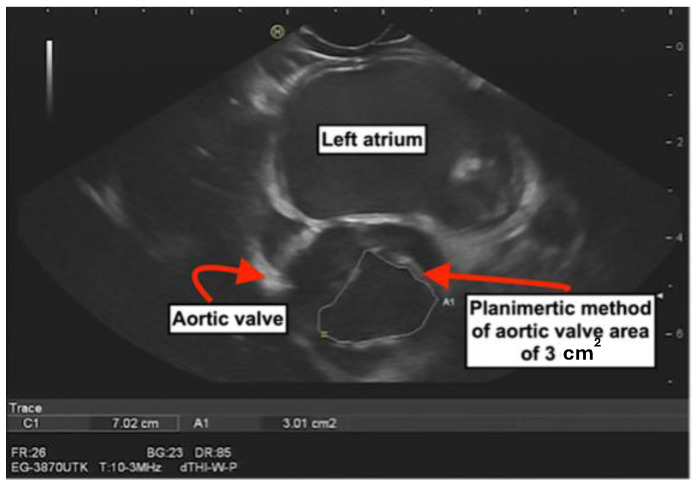
Endoscopic ultrasound demonstrated a planimetry method for the calculation of aortic valve area.

**Figure 6:**
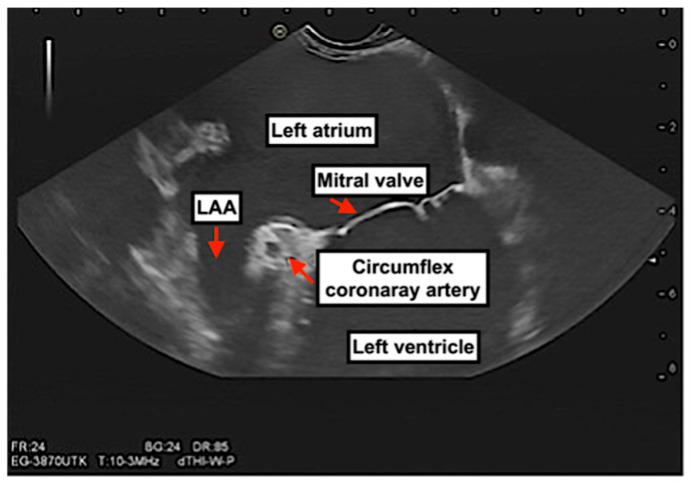
Endoscopic ultrasound demonstrated the morphology of the mitral valve, circumflex artery, and left atrial appendage.

EUS can provide high-resolution images for cardiovascular structures as follows:

1. Evaluation of aortic valve morphology, function, and vegetation (Figure 5, and Videos S1 and S2) [14];
2. Evaluation of the mitral valve’s morphology, function and vegetation, and structural mitral valve intervention, such as mitral clip (Figure 6, Videos S3 and S4). The mitral clip is a catheter-based device used for edge-to-edge mitral valve repair when treating symptomatic functional mitral regurgitation (MR) in patients at high/prohibitive surgical risk;
3. Evaluation of the tricuspid valve’s morphology and function (Video S1) and pulmonary valve (Video S5).

However, the technique does not allow for optimal visualization or assessment of cardiac valves, which appear only partially delineated and lack sufficient detail for accurate functional or morphological analysis and for guiding transcatheter procedures. The use of three-dimensional technology, multiplane, limited rotational imaging, and the absence of color and spectral Doppler capabilities need to be developed further.

### 2.5. Assessment of Cardiac Chamber Anatomy and Pathology

**Figure 7:**
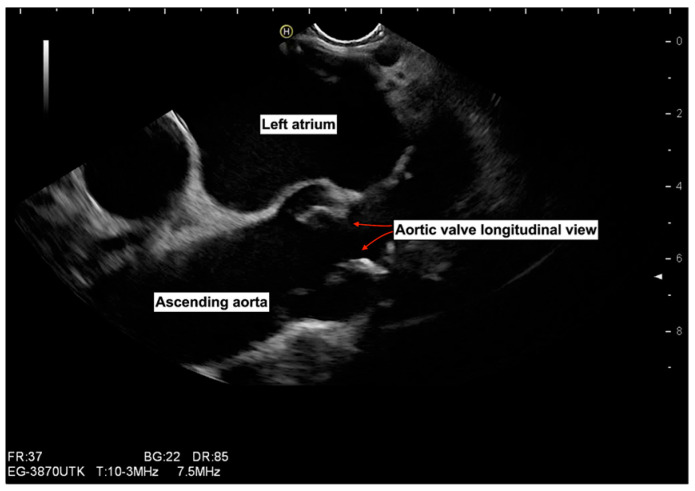
Endoscopic ultrasound demonstrated the morphology of left atrium in another view and the relation to longitudinal view of aortic valve and ascending aorta.

**Figure 8:**
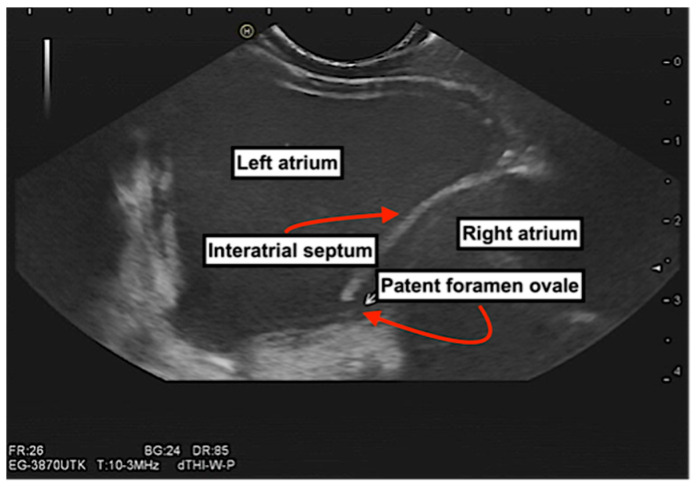
Endoscopic ultrasound demonstrated the morphology of left atrium in one view and the relation to right atrium. Patent foramen oval is incidentally detected.

**Figure 9:**
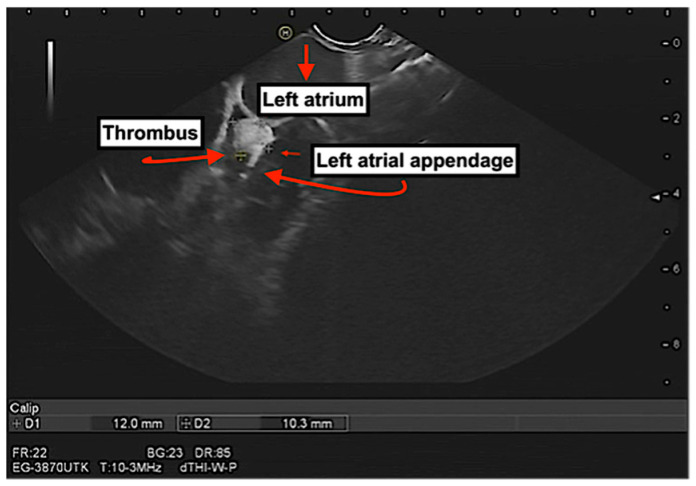
Endoscopic ultrasound demonstrated the morphology and thrombus in left atrial appendage.

**Figure 10:**
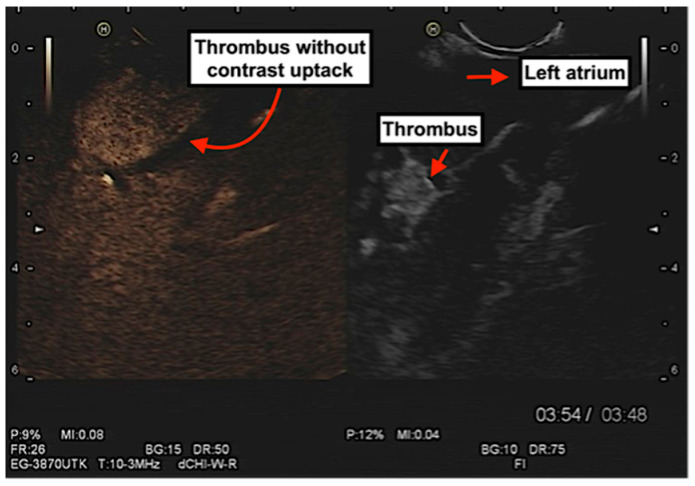
Contrast endosonographic ultrasound demonstrated the size and morphology of thrombus in left atrial appendage and its hypo-enhanced characteristic in the same patient.

**Figure 11:**
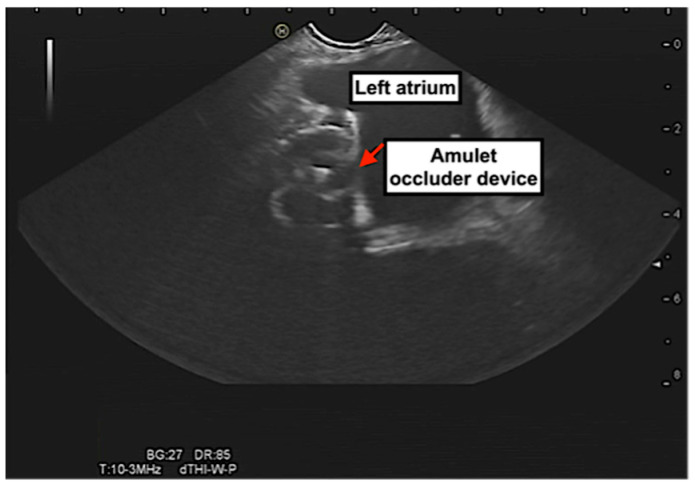
Endoscopic ultrasound demonstrated the morphology of amulet occluder device of left atrial appendage without leak, thrombus or vegetation.

**Figure 12:**
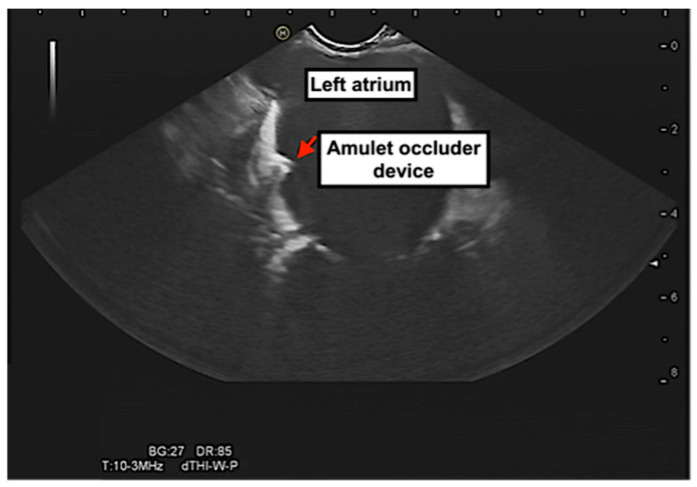
Ultrasonographic ultrasound demonstrated with another view the morphology of amulet occluder device of left atrial appendage without leak, thrombus or vegetation.

1. Assessment of the left atrium’s morphology, area, and volume (Figure 5, Figure 6 and Figure 7, and Video S6);
2. Assessment of atrial septal defects and patent foramen ovale with right/left shunt, thrombus or vegetation (Figure 8);
3. Assessment of the left atrial appendage’s (LAA) morphology, thrombus/myxoma, fibroelastoma, etc. (Figure 9 and Figure 10, and Videos S7–S9). In addition, contrast endosonographic ultrasound can be used to take images of vascularity and vessel patterns in an organ of interest, especially for those with a small volume and low-velocity blood flow. Consequently, EUS can differentiate between thrombus and myxoma, not only via their distinguishing features of size, origin, shape, mobility, and prolapse but also by using contrast enhancement. Compared with the adjacent myocardium, malignant and vascular tumors are hyperenhanced on the images, whereas stromal tumors are hypo-enhanced and thrombi are nonenhanced [21];
4. Evaluation of the left atrial appendage (LAA) occluder, device dislocation, device-related leakage, vegetation, or thrombus (Figure 11 and Figure 12). Notably, the device for the percutaneous occlusion of the LAA enacts a minimally invasive catheter-based intervention that prevents any blood clots in the LAA from entering the bloodstream. Thus, it reduces the risk of thromboembolic complications associated with non-valvular atrial fibrillation. EUS could provide adequate and precise visualizations of the LAA and complement the use of TOE in LAA evaluation. The use of EUS in this context can (1) rule out LAA thrombus, (2) ensure the device’s stability after release, (3) check for device-related leaks, (4) rule in/out device-related thrombus and vegetation, and (5) monitor complications, such as cardiac tamponade. It is important to note that TOE currently represents the standard imaging modality for LAA thrombus exclusion, pre-procedural assessment before atrial fibrillation (AF) ablation or LAA closure, and post-implant surveillance of occlusion devices. We therefore highlight the technical feasibility and demonstrate a potential clinical utility of EUS. However, clinical equivalence or interchangeability with TOE cannot be established based on anecdotal observations and limited case-based experience, and, so, more studies are required;
5. Evaluation of left ventricular function with limitation and thrombus formation (Video S10).

### 2.6. Assessment of the Aortic and Pulmonary Arteries

**Figure 13:**
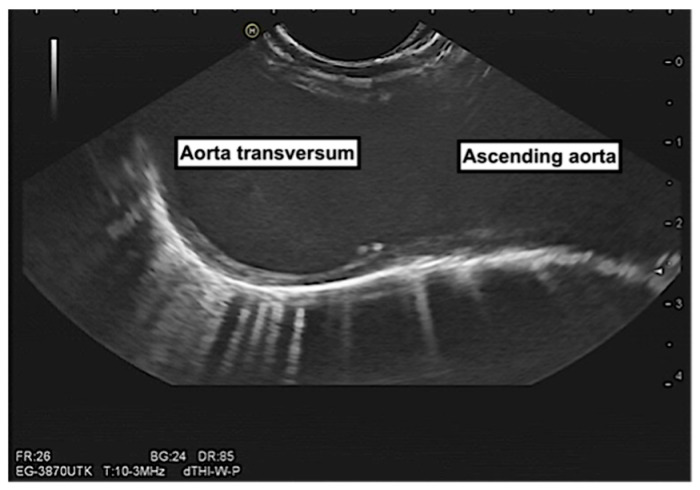
Endoscopic ultrasound demonstrated the anatomy of ascending and transversus aorta, respectively.

**Figure 14:**
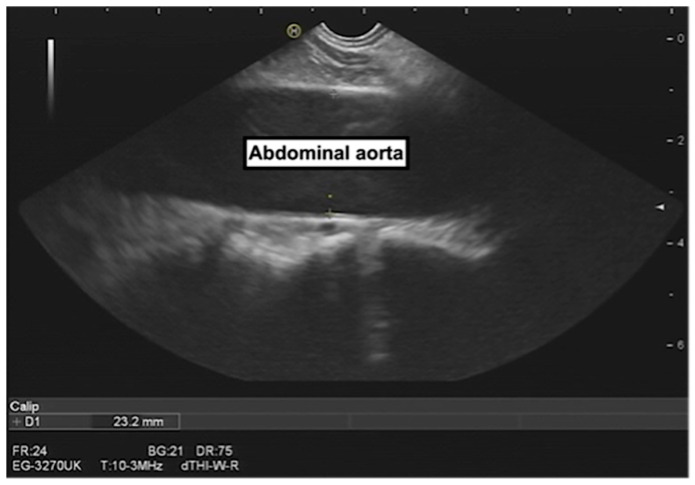
Ultrasonographic ultrasound demonstrated the anatomy of abdominal aorta.

1. Assessment of the diameter of the ascending, transverse and descending aorta, vegetation, thrombus, dissection, ulcers, aortic vascular diseases, etc. (Figure 13 and Figure 14) [14].
2. Assessment of the main stem and the right and left pulmonary arteries’ morphology/diameter and structure, embolism, pulmonary valve’s function, and pulmonary artery embolism (Video S5) [14].

### 2.7. Assessment of Pleural Effusion, and Pericardial Effusion 

**Figure 15:**
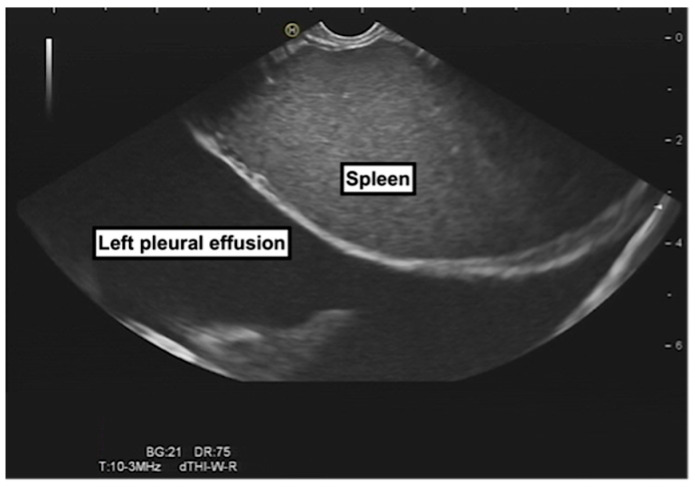
Endosonographic ultrasound demonstrated a moderate left pleural effusion.

**Figure 16:**
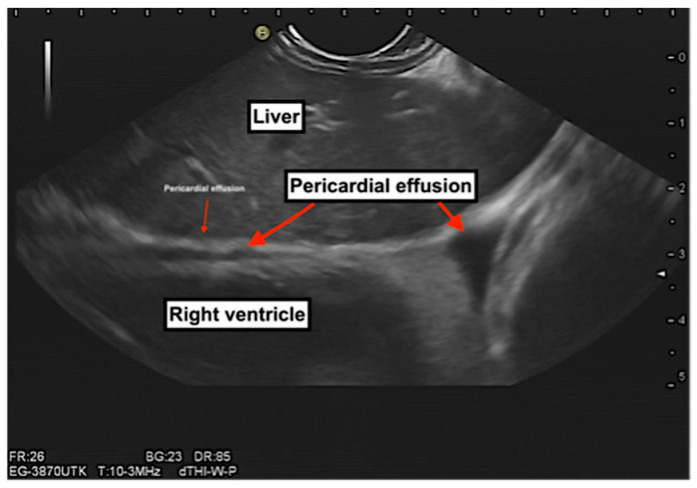
Endosonographic ultrasound demonstrated a minimal pericardial effusion.

Assessment of Pleural Effusion (Figure 15), and Pericardial Effusion (Figure 16).

### 2.8. EUS-Guided Tissue Characterization

Innovations have been made in combining high-resolution imaging with the mapping of the elastic properties and stiffness of soft tissue via sonoelastography. This methodology can help identify whether a tissue is hard or soft and can, thus, yield greater diagnostic information about disease status.

Qualitative assessments are based on the superimposition of a colored image over a conventional grayscale EUS image for a region of interest. In terms of strain level, hard tissue is colored blue, intermediate hard tissue is colored green, and soft tissue is colored red. In addition, semi-qualitative assessments using the strain ratio and strain histograms facilitate the identification of the tissues’ properties.

In this context, sonoelastography could be applied in cardiology to differentiate a hard mass (organized old thrombus or tumor) from a soft mass (soft thrombus) and to guide biopsies. More studies are required to fully elucidate the efficacy of these diagnostic tools. Furthermore, this technique allows for the selection of the most probably malignant lymph and thus helps in the selection and guidance of EUS-guided fine-needle aspiration (FNA) applied in the lymph nodes and for staging purposes in lung, cardiac, and mediastinal cancers. The main limitations of this approach are motion artifacts. The size and depth of the region of interest should remain similar when the strain rate is calculated, and the negative predictive value remains low (60–70%) [22]. Implementation of this diagnostic modality to assess cardiovascular mass, in addition to other types of multimodal imaging such as computed tomography (CT) and magnetic resonance imaging (MRI), can add diagnostic clues for differentiating benign from malignant tumors. Therefore, unnecessary operative procedures for diagnostic purposes can be avoided, along with the additional risk to vulnerable patients [23,24].

### 2.9. EUS-Guided Cardiovascular Interventions

In the last few years, the modes of use of endoscopic EUS in gastroenterology have been evolving from diagnostic to interventional procedures [25].

EUS is used to evaluate the depth of early gastrointestinal cancers and submucosal tumors (SMTs) to determine resectability. It identifies the layer of origin, surrounding vascular structures, and fibrosis, which helps predict bleeding risks and procedure time. The first step is precise mapping and saline injection into the muscularis propria to create a safety cushion for endoscopic submucosal dissection (ESD). In addition, EUS-guided gallbladder, pancreatic duct, and biliary drainage with a stent is a routine procedure in daily clinical practice. EUS is also used to drain pseudocysts and also during fluid collection. EUS-guided celiac plexus neurolysis, radiofrequency ablation, and gastric varices management are also feasible [26].

Due to the proximity of the posterior mediastinum to the esophagus, cardiovascular structures can be technically assessed using the EUS, and a biopsy or minimally invasive interventions can be performed. This methodology could be used to perform real-time sampling and confirm the diagnosis of rare primary cardiac tumors in place of open-heart surgery in high-risk patients. In animal models, samples of the coronaries, atria, ventricles, and valvular apparatus were taken with no major adverse events in isolated cases [27].

### 2.10. Observational Studies and Case Reports

There are a few case reports on the use of EUS as an interventional diagnostic tool to assess cardiovascular structures in high-risk, non-operable patients.

A case of a 65-year-old woman with cardiac CT involved a right-sided cardiac tumor with signs of invasion of the interatrial septum, superior and inferior caval veins, and liver parenchyma. An attempt at radiologically guided puncture and biopsy was unsuccessful.

Thus, a second attempt at EUS-guided transesophageal puncture was made using a linear echoendoscope, which was introduced into the middle thoracic esophagus, and a solid hypoechoic mass attached to the atrial right wall was observed. The lesion was punctured with a 22-gauge needle (Expect, Boston Scientific, Natick, MA, USA) via smooth backward and forward movements. The cytopathology confirmed the diagnosis of cardiac angiosarcoma [28].

In another case, a 26-year-old male presented with recent-onset dyspnea. Cardiac magnetic resonance imaging showed a large infiltrative mass, which post-contrast enhancement revealed to be in the right atrium with pericardial extension.

Linear EUS revealed a large mass (8 × 8 cm) within the right atrium, with diffuse thickening in the inferior and posterior regions.

A 25-gauge core needle (Acquire, Boston Scientific Co., Marlborough, MA, USA) was inserted from the posterior pericardium via two smooth forward and backward movements. The cytopathology results derived on site confirmed the diagnosis of cardiac angiosarcoma [29].

A case of successful EUS-guided transesophageal pericardial cyst drainage has also been reported [30].

Two case reports describe successful endoscopic ultrasound with bronchoscope-guided fine-needle aspiration (EUS-B-FNA) of the left atrial masses, undertaken in patients for whom cardiac surgery was considered hazardous due to comorbidities or previous surgical interventions. Cytopathology confirmed the diagnosis of Burkitt lymphoma and synovial sarcoma [31].

These findings suggest that, in selected cases, linear EUS is feasible as a minimally invasive technique for intracardiac tumor diagnostics, particularly in vulnerable non-operable high-risk patients. It is important to note that EUS-guided interventions are a well-established daily clinical practice in gastroenterology. In contrast, the available literature on EUS-based cardiovascular interventions is limited. The absence of prospective clinical data and the potential procedural risks with high mortality risk associated with transesophageal cardiovascular puncture and biopsy, such as atrioesophageal fistula formation, should be further assessed in a large cohort.

### 2.11. Interdisciplinary Teamwork

EUS can strengthen interdisciplinary teamwork in management strategies. The following scenarios are examples from daily clinical practice.

- Patients with gastrointestinal tumors are immunocompromised and have a higher risk of pulmonary embolisms, ischemic events, and infective endocarditis. Routine cardiovascular investigation, particularly in high-risk patients, during gastrointestinal EUS investigations could help detect these pathologies earlier. This could, therefore, help in the commencement of an effective and timely management strategy, preventing repeated investigations;
- The secondary pain experienced by tumor patients could mask and overshadow the symptoms of other pathologies, such as pulmonary embolism;
- Upper gastrointestinal pain may occur due to angina pectoris/aortic dissection or a peptic ulcer;
- Patients under oral anticoagulation treatments with gastrointestinal bleeding are generally admitted to internal medicine and not the cardiology department. A device for LAA closure reduces the dose of anticoagulation, the rates of gastrointestinal bleeding events, and drug–drug interactions;
- Fevers of unknown origin could develop due to infective endocarditis or abscess formation;
- Gastrointestinal ischemic or thromboembolic events could be of primary cardiac origin;
- Dyspnea of unknown origin could result from a pulmonary embolism or pulmonary artery sarcoma. An EUS or endobronchial ultrasound (EBUS) could be used to differentiate and identify these structures. EUS-guided biopsy may facilitate amyloid and sarcoid diagnosis;
- Intracardiac structures can be identified and evaluated for the development of further differential diagnoses, such as intracardiac thrombus, myxoma, and tumors, using EUS with contrast;
- Heyde syndrome is a multisystem disorder characterized by the triad of aortic stenosis (AS), gastrointestinal bleeding, and acquired von Willebrand syndrome [32].

These scenarios highlight the necessity of continuous interplay between the daily clinical practice of different subspecialties to improve outcomes in populations of advanced age with more complex comorbidities.

## 3. Discussion

EUS is recommended for use in the current guidelines for cancer management, as it provides images of the highest possible resolution and facilitates detailed tissue characterization during the local staging of gastrointestinal tumors [2], showing a broader applicability of elastography across various echocardiographic domains.

EUS’s precision in targeting inserting a needle into adjacent organs—a well-established procedure in daily gastrointestinal interventions—could facilitate the performance of transesophageal extra- and intracardiac interventions under direct EUS guidance [26].

The introduction of EUS in CVD may also help management in high-risk, vulnerable, and non-operable patients by facilitating both the diagnosis of the underlying pathology and minimal therapeutic intervention through fine-needle aspiration biopsy. However, more large studies are still required to assess the safety and efficacy of EUS applications in CVD, as the current evidence is limited to a few case reports.

Thanks to the collaboration between Hitachi and Pentax over the last decade in developing the HI-VISION Preirus Ultrasound system [33], electronic radial and linear EUS scopes have allowed for a significant improvement in image quality due to improved resolution, delineation, the blending of contrast, and depth of imaging while reducing noise. Such innovations increase the potential of more diagnostic and therapeutic EUS-based applications being developed [34].

The main strengths of EUS and its potential applicable role in cardiology can be summarized as follows:

Its higher resolution means it provides a more precise ideal high-resolution imaging modality for the accurate identification of cardiovascular structures and pathology, which is key to management and improving outcomes [35]. This practice could complement TOE.

The introduction of the endoscope, aided by direct visualization of gastroesophageal anatomy using an endoscopic camera, could reduce procedural complications. This may be of benefit in patients with contraindications for TOE due to a high risk of esophageal bleeding or complications that may require immediate intervention.

We would like to point out that miniaturized pediatric probes are currently available as an alternative in patients with contraindications to conventional TOE. These offer an imaging quality comparable to standard adult TOE probes and may provide a feasible alternative in selected cases [7].

- Contrast-enhanced EUS imaging is used to elucidate vascularity and vessel patterns in the organ of interest, especially for those with a small volume and low-velocity blood flow. It also aids in the differential diagnosis of focal masses via not only their different size, origin, shape, mobility, and prolapse characteristics but also via contrast enhancement. The recent development of low-mechanical-index contrast harmonic EUS imaging helps in improving the diagnosis, staging, and monitoring of antiangiogenic treatment [21];
- Sonoelastography helps to map the elastic and stiffness properties of soft tissue and to differentiate lymph nodes (LNs). For example, benign lymph nodes have a homogenous and red appearance, whereas malignant LNs are colored blue. Thus, they can be targeted with a needle to confirm the presence of focal hard malignant infiltration up to 3–5 mm in the blue area, itself inside an otherwise normal homogenous soft LN (red) area [36];
- These applications highlight the importance of interdisciplinary teamwork in the development of diagnosis and treatment strategies;
- In addition, the combination of fine-needle aspiration with excellent image quality allows the endoscope to be used for a wide range of procedures and could facilitate EUS-based cardiovascular sampling and intervention. Therefore, it helps in confirming diagnoses and constitutes a new, exciting, and expanding area. It could further help in precluding unnecessary explorative surgical interventions.

### Current Technical Limitations

Although such reports are promising, they are only anecdotal, and these advanced imaging tools have not yet been investigated for comprehensive cardiac evaluation beyond the characterization of tissue masses.

In addition, EUS requires more time for sterilization, preparation, and investigation, and its main disadvantage is its high cost. While elastography and contrast-enhanced ultrasound may provide additional diagnostic information, their application is currently most relevant in the evaluation of cardiac and extracardiac masses. In its present form, the technique does not allow for optimal visualization or assessment of cardiac valves, which appear only partially delineated and lack sufficient detail for accurate functional or morphological investigations or for guiding transcatheter procedures. The use of three-dimensional technology, limited multiplane imaging capabilities, limited rotational imaging, the absence of color and spectral Doppler capabilities, and the absence of robust cardiovascular validation studies remain areas that need to be developed further [34].

Therefore, the current clinical applications are limited, and EUS cannot be an alternative to standard techniques. However, it remains a promising technique for the future, pending technical advancements and refinement.

Finally, we hereby open a new field in the literature, highlighting the advantages of EUS as a gold standard imaging modality for tumor assessment and providing fundamental anatomical knowledge for future perspectives and implications.

More studies are required to assess the safety and efficacy of such interventions, comprehensively compare this procedure with other cardiac imaging modalities, and determine the exact applications and benefits of using EUS in cardiovascular medicine as a diagnostic and cardiovascular-based intervention imaging modality.

## 4. Conclusions

The introduction of endoscopic ultrasound into cardiology enables real-time high-resolution assessments of cardiovascular anatomy and pathology. Endoscopic ultrasound differs in terms of the design of its US tip, the contrast-enhanced EUS imaging, fine-needle aspiration, and mapping of the elastic and stiffness properties of soft tissue.

The combination of fine-needle aspiration with excellent image quality allows the endoscope to be used for a wide range of procedures and may facilitate promising future applications of EUS-based cardiovascular intervention.

## 5. Future Perspectives

The introduction of endoscopic ultrasound into cardiology enables real-time high-resolution assessments of cardiovascular anatomy and pathology during routine gastrointestinal procedures, reduces the risk of esophageal trauma arising from the introduction of a probe with a high-resolution camera to visualize anatomy, and may complement TOE.

Until stronger evidence emerges, the use of EUS in CVD should be individualized—balancing potential benefits against device-related risks. More studies are required to assess the safety and efficacy of EUS as a diagnostic and EUS-based intervention in CVD, comprehensively compare this procedure with other cardiac imaging modalities, and determine its exact applications in CVD.

While elastography and contrast-enhanced ultrasound may provide additional diagnostic information, their application is currently the most relevant in the evaluation of cardiac and extracardiac masses. The current clinical applications are limited, and EUS remains a promising technique for the future, pending technical advancements and necessitating a continuous interplay between daily clinical practice and industry.

## Figures and Tables

**Table 1:** The main differences between transesophageal echocardiography and endosonographic ultrasound.

Type of Ultrasound	Transesophageal Echocardiography	Endoscopic Ultrasound
Ultrasonic functions		
Frequency	Between 2 and 7 MHz [3]	5, 6.5, 7.5, 9, 10 and up to 13 MHz [4]
Frame rate	30 fps [5]	22 fps [6]
Scanning angle	180° [7]	120–180° [4]
Depth of field	2–30 mm [7]	5–100 mm [4]
Three-dimensional function	Yes [7]	A 3D-based model based on registered preoperative data is underway [8]
Endosonographic imaging	Depends on: Temporal resolution; Lateral resolution; Longitudinal resolution; Axial resolution; Different frequencies; Different focus [9].	Depends on: Use of radial array EUS for diagnostic purposes; Use of linear array EUS for diagnostic and therapeutic purposes.
Endoscopic image resolution		
	Charge-coupled device (CCD) chips not available	A generally higher resolution, up to a million pixels; Standard-definition endoscopes are fitted with CCD chips that produce image signals with a resolution of 100,000 to 400,000 pixels [10].
Probe design		
	Phased array [7]	Convex radial and linear array [11]
	Ultrasound	Ultrasound and endoscope
Working channels		
	Only an image transmission system [7]	A lightning system; A channel for suction and for air; A channel for the ingress of water to clean the lens and the insertion of biopsy forceps, or needles in the case of longitudinal EUS; A channel with a bending mechanism to enable the deflection of the endoscope’s tip; An image transmission system [12].
Image rotation capacity	Yes [7]	No
Guiding the probe via direct visualization	No	Under direct sonographic and endoscopic visualization [4]
Procedure		
Purposes	Diagnostic	Diagnostic and intervention in the case of longitudinal EUS
Visible anatomical structures	Almost all cardiac structures [13]	Cardiac structures with limitations in terms of right-side structures, and the assessment of different valvular planes [14]
Duration of investigation	More examinations can be performed per time unit	More time required for preparation and investigation
Complication rate	Ranges from 0.2% to 0.5% [15]	Lower, at a rate of 0.15% [16]
Sterilization	Requires about 20 min in sterilizing fluid [7]	Must go through a sterilization machine, which takes longer Automated Endoscope Preprocessor (AER) machines take less time [17]
Cost	Lower [7]	Higher [4]

## Data Availability

The datasets used and all clinical and investigation images and laboratory results during the current study, are available from the corresponding author upon reasonable request.

## Supplementary Materials

The following supporting information can be downloaded at https://www.mdpi.com/article/10.3390/jcm15135006/s1: Table S1: The main features of endosonographic ultrasound EG-3870TK and EG-3270UK [28]. Table S2: The main features of the Fujifilm EG-740UT curved linear endosonographic ultrasound scope. Table S3: Summarizes the main features of the TGF-UC180J linear ultrasound endoscope. Video S1: Aortic and tricuspid valves in the endoscopic ultrasound. Video S2: Aortic valve endocarditis in long-axis view in the endoscopic ultrasound. Video S3: Mitral valve in 2-chamber view in the endoscopic ultrasound with infra-valvular vegetation. Video S4: Mitral valve with mitral clip in the endoscopic ultrasound. Video S5: Pulmonary valve in the endoscopic ultrasound. Video S6: Left atrial appendage and circumflex coronary artery in the endoscopic ultrasound. Video S7: Left atrium in the endoscopic ultrasound. Video S8: Left atrium demonstration in another view in the endoscopic ultrasound. Video S9: Left atrial appendage with thrombus in the contrast endoscopic ultrasound. Video S10: Left ventricle demonstration in the endoscopic ultrasound. Video link: https://drive.google.com/drive/folders/1Iik1N3P6sji-zv7vj0YYFivMj6V-1fQD?usp=drive_link (accessed on 18 March 2026).

## Abbreviations

CT	Computed tomography
CVD	Cardiovascular disorders
ESD	Endoscopic submucosal dissection
EUS	Endoscopic ultrasound
EUS-B-FNA	Bronchoscope-guided fine-needle aspiration
FNA	Fine-needle aspiration
LAA	Left atrial appendage
MR	Mitral regurgitation
MRI	Magnetic resonance imaging
SMTs	Submucosal tumors
TOE	Transesophageal echocardiography
US	Ultrasonography
