# Supplementary material for: The Role of Endoscopic Ultrasound in Cardiology: Clinical Applications and Future Perspectives, a New Era of Minimally Invasive Cardiovascular Diagnosis and Intervention
Source: J Clin Med. 2026 Jun 26;15(13):5006. doi: 10.3390/jcm15135006 (PMC13362660; doi:10.3390/jcm15135006)
Supplement: Supplementary file 1 [file jcm-15-05006-s001.zip › jcm-4329021-supplementary.pdf]

Supp. Table S1: The main features of endosonographic ultrasound EG-3870TK and EG-3270UK [28].

Model Name	EG-3870UTK	EG-3270UK
Optical system		
Field of view	120° (45° forward oblique)	120° (50° forward oblique)
Depth of field	5-100 mm	
Bending capability U/D/R/L	130°/130°/120°/120°	130°/130°/120°/120°
Rigid distal width		
Probe	12.5 x 12.3 mm	∅ 11.5 mm
Optical	∅ 14.3 mm	∅ 12 mm
Insertion section		
Distal end width	∅ 14.3 mm	∅ 12 mm
Insertion tube width	∅ 12.8 mm	∅ 10.8 mm
Maximum insertion portion width	∅ 14.65 mm	∅ 12.5 mm
Maximum instrument channel width	∅ 3.8 mm	∅ 2.8 mm
Insertion portion working length	1,250 mm	
Total length	1,560 mm	
Ultrasound function		
Acoustic frequency	5-10 MHz switchable	
Scan direction	Linear	
Scan method	Convex	
Scan angle	120°	
Balloon	OF-A67—Removable	OE-A56—Removable
Laser treatment	Incompatible	
Electrosurgery treatment	Compatible	

Supp. Table S2: The main features of the Fujifilm EG-740UT curved linear endosonographic ultrasound scope.

Endoscopic functions of Fujifilm EG-740UT curved linear EUS scope [5]	
Optical system	
Viewing direction	40°
Observation range	3-100 mm
Field of view	140°
**Insertion section**	
Distal end ø	14.5 mm
Insertion tube ø	12.6 mm
Bending capability U/D/R/L	150°/100°/100°/100°
Working length	1,250 mm
Total length	1,550 mm
Working channel	4.0 mm
**Ultrasonic functions**	
Scanning method	electronic curved linear array scan
Scanning angle	180°
Fujifilm compatible balloon	BS-102

Supp. Table S3: summarizes the main features of the TGF-UC180J linear ultrasound endoscope.

Main features of the TGF-UC180J linear ultrasound endoscope [6]	
**Optical system**	
Field of view	100°
Direction of view	Forward oblique viewing 50°
Depth of field	3-100 mm
**Insertion section**	
Distal end outer diameter	ø 13.4 mm
Distal end enlarged	• Light guide lens<br>• Air/water nozzle<br>• Objective lens<br>• Instrument channel outlet<br>• Ultrasound transducer<br>• Balloon aspiration port<br>• Balloon water feeding port
Insertion tube outer diameter	ø 10.9 mm
Insertion section working length	1,250 mm
**Instrument channel**	
Channel inner diameter	ø 2.2 mm
Minimum visible distance	5 mm
Airflow Rate	20 cm3/s
Bending range capability U/D/R/L	130°/90°/90°/90°
Total length	1,563 mm
**Ultrasound function**	
Transducer surface maximum temperature	<43 °C
Ultrasound method	Electronic radial array
Ultrasound direction	Perpendicular to the insertion direction
Frequency	5/6/7.5/10/12 MHz
Scanning Range	360°
Contact method	Balloon method, sterile deaerated water immersion method
Ultrasound function with EU-ME2 Premier Plus operation mode	B-mode, THE mode, H-Flow mode, color flow mode, power flow mode, PW mode, CH-EUS mode, and ELST mode (elastography)

Video legends

Video S1: Aortic and tricuspid valves in the endoscopic ultrasound.
Video S2: Aortic valve endocarditis in long axis view in the endoscopic ultrasound.
Video S3: Mitral valve in 2-chamber view in the endoscopic ultrasound with infra-valvular vegetation.
Video S4: Mitral valve with mitral clip in the endoscopic ultrasound.
Video S5: Pulmonary valve in the endoscopic ultrasound.
Video S6: Left atrial appendage and circumflex coronary artery in the endoscopic ultrasound.
Video S7: Left atrium in the endoscopic ultrasound.
Video S8: Left atrium demonstration in another view in the endoscopic ultrasound.
Video S9: Left atrial appendage with thrombus in the contrast endoscopic ultrasound.
Video S10: Left ventricle demonstration in the endoscopic ultrasound.
https://drive.google.com/drive/folders/1Iik1N3P6sji-zv7vj0YYFivMj6V-1fQD
